# Expressive visual text-to-speech as an assistive technology for individuals with autism spectrum conditions

**DOI:** 10.1016/j.cviu.2015.08.011

**Published:** 2016-07

**Authors:** S.A. Cassidy, B. Stenger, L. Van Dongen, K. Yanagisawa, R. Anderson, V. Wan, S. Baron-Cohen, R. Cipolla

**Affiliations:** a Centre for Psychology, Behaviour and Achievement, Coventry University, Coventry CV1 5FB, UK; b Toshiba Research Europe Ltd., 208 Science Park, Cambridge CB4 0GZ, UK; c Autism Research Centre, Department of Psychiatry, University of Cambridge, Douglas House, 18B Trumpington Road, Cambridge CB2 8AH, UK; d Maastricht University, Faculty of Psychology, Maastricht 6200 MD, The Netherlands; e Cambridgeshire and Peterborough Foundation NHS Trust, CLASS Clinic, UK; f Engineering Department, University of Cambridge, Cambridge CB2 1PZ, UK

**Keywords:** Autism spectrum conditions, Emotion recognition, Social cognition, Intervention, Assistive technology

## Abstract

Adults with Autism Spectrum Conditions (ASC) experience marked difficulties in recognising the emotions of others and responding appropriately. The clinical characteristics of ASC mean that face to face or group interventions may not be appropriate for this clinical group. This article explores the potential of a new interactive technology, converting text to emotionally expressive speech, to improve emotion processing ability and attention to faces in adults with ASC. We demonstrate a method for generating a near-videorealistic avatar (XpressiveTalk), which can produce a video of a face uttering inputted text, in a large variety of emotional tones. We then demonstrate that general population adults can correctly recognize the emotions portrayed by XpressiveTalk. Adults with ASC are significantly less accurate than controls, but still above chance levels for inferring emotions from XpressiveTalk. Both groups are significantly more accurate when inferring sad emotions from XpressiveTalk compared to the original actress, and rate these expressions as significantly more preferred and realistic. The potential applications for XpressiveTalk as an assistive technology for adults with ASC is discussed.

## Highlights

• We show a videorealistic avatar, XpressiveTalk, which speaks in various emotional tones.
• The use as an assistive technology for people with autism spectrum conditions (ASC) is discussed.
• We carry out emotion-recognition and preference studies.
• Adults with ASC are less accurate than controls, but above chance levels for inferring emotions.

## 1 Introduction

Autism spectrum conditions (ASC) are characterised by difficulties in social communication alongside unusually restrictive, repetitive behaviours and interests [1]. A key difficulty experienced by individuals with ASC, and part of current diagnostic criteria, is interpreting others’ emotions and responding appropriately [1]. Indeed, [2] originally described ASC as a difficulty with “affective contact”. Hence, a number of intervention programs aiming to improve social and communication skills in ASC, have focused on improving ability to interpret others’ emotions [3], [4], [5], [6].

Improving ability to interpret emotions in realistic social situations in people with ASC is challenging, because the intervention needs to generalize to a variety of real life social situations. New interactive technologies provide a very promising form of intervention which could improve emotion processing in real life situations for a number of reasons. First, individuals with ASC prefer interventions which involve interacting with technology rather than face-to-face or group based work, that could cause anxiety [3], [4]. Use of a computer to display emotions, instead of a face to face encounter, could therefore encourage attention to important social cues. Hence, use of technology as an intervention tool in people with ASC is particularly appealing and accessible for this clinical group. Second, interactive technologies enable people with ASC to actively experiment in safe, controlled and predictable environments repeatedly. The difficulty levels of the intervention, gradually getting more complex, can be slowly widened, and even controlled by the participant. This would provide adults with ASC a series of predictable, controllable and therefore low anxiety learning opportunities, which would not otherwise be available to these individuals in the real world. This also enables a systematic approach to learning, which is particularly in tune with the cognitive style in ASC [7].

Previous attempts to utilize technology to improve emotion recognition skills in children and adults with ASC have shown some success. For example, *The Transporters* [6] and *Mindreading* [5] interventions aim to capitalize on the strong abilities that children and adults with ASC show in constructing patterns and systems from their environment. In the case of *The Transporters*, children with ASC aged 4–7 years old passively watch trains with real human faces interact in a number of social situations over a period of 4  weeks. Post-intervention, the children with ASC reached typical control levels of emotion recognition, and training transferred to new situations not included in the original intervention videos [6]. There was also some anectodal evidence that children showed increased eye contact and interest in people post-intervention. Similarly, in the case of the *Mindreading* intervention, adults with high functioning ASC interacted with a comprehensive library of 412 naturalistic emotions in the face and voice separately, and combined, over 10–15 weeks. Adults with ASC showed improvement in their ability to recognize the emotions included in the original intervention, but this training did not transfer to other emotions or new situations [5]. Other examples come from robotic systems such as FACE which is capable of producing basic emotion expressions (e.g. happy, sad) [8]. A 20 min therapy session has been shown to elicit spontaneous eye contact and social imitation in children with autism [8]. A range of other studies also demonstrate the potential of socially assistive robots for improving eye contact and social interaction skills in children with autism [9]. However, complex natural facial expressions that present difficulties for people with ASC in everyday life are challenging to simulate using robotics.

The challenge of improving ability to interpret emotions in realistic social situations in people with ASC is for improvement to generalize beyond the scope of the original intervention, to new emotions and situations. One promising approach is for the intervention to be flexible, allowing for different levels of difficulty, and for the person undergoing the intervention to experiment and interact in the environment. With *The Transporters, Mindreading* and *FACE robotics* interventions, this was not possible.

New interactive technologies provide an opportunity for ASC individuals to practice their communication skills. In the current study we explore the scope for expressive visual speech animation as a potential intervention tool to improve emotion processing skills in adults with high functioning ASC. The technology, named XpressiveTalk, provides a near-realistic animation with dynamic emotion expressions. Previous studies of emotion processing have used animations which are highly unrealistic, e.g. [10], [11]. However, adults with high functioning ASC tend to have difficulty processing naturalistic emotions. Hence, in order to improve attention and emotion recognition in everyday life, interventions must use realistic and flexible stimuli. The benefit of XpressiveTalk as a potential intervention tool is the development of a near-realistic visual interface, which approximates the type and complexity of emotions encountered in everyday life. In order to build a realistic visual interface, face and speech models are trained based on a corpus of video recordings of an actress.

The following section provides further background from ASC research, motivating the need for generating nuanced speech and vision cues. Subsequently we provide details on the creation of the face model. We present user studies in which we first explore how adults with ASC and typically developing adults are able to infer emotions from recorded and synthesised emotions. Second, we explore how these individuals rate their preference and realism of real and synthesised emotions. These results will provide valuable insights into how adults with ASC interact with XpressiveTalk, and its potential as an intervention to improve emotion processing in these individuals.

## 2 Prior ASC research

Results from lab experiments have not consistently demonstrated emotion recognition difficulties in people with ASC, particularly high functioning adults with ASC who have verbal and intellectual ability in the average or above range [12], [13], [14]. These results are incommensurate with these individuals’ difficulties in everyday life [1]. However, recent research has shown subtle emotion recognition difficulties in high functioning adults with ASC, when interpreting emotions in realistic social situations [15], particularly when these are dynamic, and include vocal cues [16], [17], [18]. In contrast, studies that utilise static expressions posing a single emotion at high intensity, or use cartoon-like animations do not tend to show differences in emotion processing ability between those with and without ASC [19], [20], [21], [22], [23], [24]. Thus, complex stimuli which mimic the demands of emotion processing in everyday life are more likely to reveal emotion recognition difficulties in adults with high functioning ASC [16].

These results have recently been explained by difficulties processing emotions of low signal clarity in people with ASC [16]. Signal clarity is high when a single emotion is presented at high intensity, and is low when more than one emotion is presented (e.g. smiling in confusion), and in cases where facial expression and vocal cues are contradictory (e.g. saying thank you with a grimace) [25]. In everyday life, mixed emotion responses of low signal clarity tend to be expressed, such as smiling in frustration [26], happily or angrily surprised [27], or feigning a positive response to a social interaction partner [15], [28].

As these examples demonstrate, there are two important abilities necessary to interpret emotional responses of low signal clarity typically encountered in realistic social situations. First, one must be able to integrate a variety of different visual cues from the mouth and eyes. Second, one must be able to process visual and vocal information simultaneously. Adults with ASC tend to have difficulty with both these aspects of processing. For example, adults with ASC have difficulty interpreting negative [21], [24], [29] and feigned positive emotions [30] which involve integrating different cues from the mouth and eyes, and mixed emotions (e.g. happy and surprised) [31]. Second, children with ASC are less susceptible to the McGurk effect (a phenomenon in speech perception based on interacting speech and vision cues), tending to report the vocally produced syllable, rather than automatically integrating visual cues and reporting a blend of the two information channels [32]. Adults with ASC also appear to rely more on speech content, rather than integrating non-verbal cues when interpreting complex emotions from videos of social interactions [18], spontaneous emotional responses [15], [16], and when distinguishing consistent from inconsistent facial and vocal emotions [10].

Difficulties integrating visual cues, and tendency to rely on speech content in people with ASC, could be due to reduced attention to social information. A key early indicator of ASC in infants is lack of eye contact and following others’ gaze [33], [34], [35], [36]. Research utilising eye tracking technology while viewing social and emotional stimuli have shown that people with ASC look less to social information, such as people, eyes and faces [37], [38]. In high functioning individuals with ASC, differences in attention to social information is most pronounced in the first few seconds of viewing time [39], [40], [41], [42], or when stimuli are dynamic and complex (i.e. involving more than one person) [16], [43]. Research has also suggested that attention to social information, such as the eyes in people with ASC, causes aversive over-arousal, and is thus actively avoided by these individuals [21].

Clearly, adults with ASC have difficulties processing emotions of low signal clarity, involving integration of complex and sometimes contradictory visual and vocal information. Lack of attention to social information (eyes and people) could be a key contributor to these difficulties. Infants who show reduced social attention tend to be diagnosed with ASC later on. This demonstrates the importance of social attention skills in the development of ASC [36], [37].

## 3 Expressive visual text-to-speech

In this section we present a method for generating a near-videorealistic avatar. Given an input text, the system is able to produce a video of a face model uttering the text. The text can be annotated with emotion labels that modulate the expression of the generated output. The system is trained on a large corpus containing speech and video recordings of an actress.

### 3.1 Visual text-to-speech (TTS)

Text-to-speech (TTS) synthesis systems generate computer-synthesised speech waveforms corresponding to any text input. A TTS system is typically composed of a front-end and a back-end. The front-end takes as input a string of text and converts it into a sequence of phonemes and a linguistic specification consisting of context features describing the linguistic and phonetic environment in which each phoneme occurs. The back-end then takes these context features to generate a waveform. A conventional approach called unit-selection TTS re-used existing segments in the training database that matched best the phonetic contexts required and concatenated them at synthesis time. More recently, statistical parametric approaches have become more widely used. Instead of selecting actual instances of speech from a database, in statistical parametric approaches such as HMM (hidden Markov model) based TTS [44], parametric representations of speech are extracted from the speech database and are modelled by a set of models such as HMMs. Concatenating the HMMs produces a set of parameters which can then be resynthesised into synthetic speech. Since it is not practical to collect a training database that covers all possible linguistic contexts, decision trees are used to cluster similar environments [45]. For any given input context, the means and variances to be used in the HMMs may be looked up using the decision tree. We extend this TTS method to visual TTS by concatenating the audio feature vector with a video feature vector so the HMMs generate a temporal sequence of parameters that are synthesised into a speech and video signal.

### 3.2 Cluster adaptive training (CAT)

One of the advantages of HMM–TTS is its controllability. Unlike unit-selection, HMM–TTS allows easily synthesising contexts which are not found in the training database. This offers the possibility to achieve expressive TTS without requiring large expression-dependent databases, and to synthesise new expressions. For the current study, Cluster Adaptive Training (CAT) [46] was used to achieve expressive TTS.

**Fig. 1:**
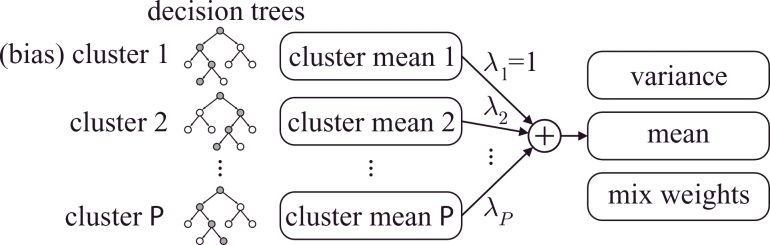
Cluster adaptive training (CAT). Each cluster is represented by a decision tree and defines a basis in expression space. Given a position in this expression space defined by $\lambda^{\mathrm{expr}}=\left[\lambda_{1}...\lambda_{P}\right]$ the properties of the HMMs to use for synthesis can be found as a linear sum of the cluster properties.

CAT is an extension to HMM–TTS, which uses multiple decision trees to capture speaker- or emotion-dependent information. Fig. 1 shows the structure of the CAT model. Each cluster has its own decision tree, and the means of the HMMs are determined by finding the mean for each cluster and combining them using the formula:

(1) $$\mu_{m}^{\mathrm{expr}}=M_{m}\lambda^{\mathrm{expr}},$$

where $\mu_{m}^{\mathrm{expr}}$ is the mean for a given expression, *m* is the state of the HMM, **M**_*m*_ is the matrix formed by combining the means from each cluster and ***λ***^expr^ is a weight vector.

Each cluster in CAT may be interpreted as a basis defining an expression space. To form the bases, each cluster is initialised using the data of one emotion (by setting the ***λ***’s to zero or one as appropriate). The Maximum-Likelihood criterion is used to update all the parameters in the model (weights, means and variances, and decision trees) iteratively. The resulting ***λ***’s may interpreted as coordinates within the expression space. By interpolating between $\lambda^{{\mathrm{expr}}_{1}}$ and $\lambda^{{\mathrm{expr}}_{2}}$ we can synthesise speech with an expression combining two of the originally recorded expressions. Since the space is continuous, it is possible to synthesise at any point in the space and generate new expressions. More details are described in [47].

### 3.3 Training the XpressiveTalk system

**Fig. 2:**
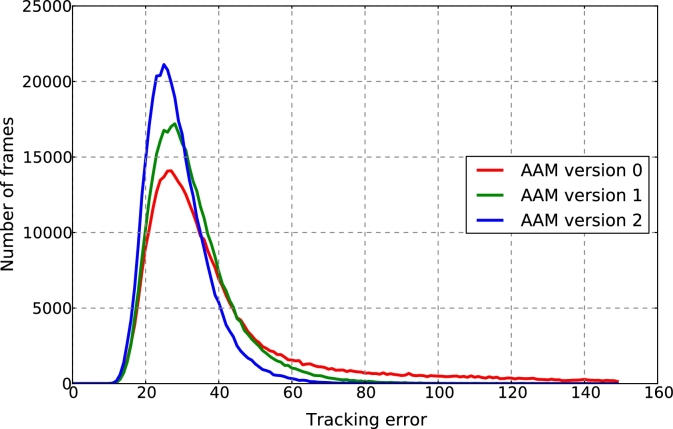
Error histograms for three iterations of the model building process. Errors are decreased with each new iteration of the model.

**Fig. 3:**
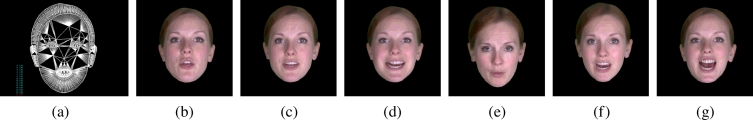
Active Appearance Model. The shape mesh is shown in (a). Example synthesis results for (b) neutral, (c) tender, (d) happy, (e) sad, (f) afraid and (g) angry.

Our training corpus comprised 6925 sentences, capturing six emotions: neutral, tender, angry, afraid, happy, and sad. The speech data was parameterised using a standard feature set consisting of 45 dimensional Mel-frequency cepstral coefficients, log-F0 (fundamental frequency) and 25 band aperiodicities, together with the first and second time derivatives of these features. The visual data was parameterised using an Active Appearance Model (AAM) with specific improvements for face synthesis. The improvements include pose-invariance, region-based deformations, and textures for the mouth region [48]. In the following we describe the training procedure of the model. To build an AAM a small initial set of training images is labelled with a set of keypoints marking the same location of the face in each image. The initial set consists of images selected for each of the following sounds in each emotion: (1) *m* in *man*, (2) *ar* in *car*, (3) *ee* in *eel*, (4) *oo* in *too*, (5) *sh* in *she*. The initial AAM is then tracked over the whole training corpus (≈ 10^6^ frames) using the method in [49]. Poorly reconstructed frames are added to the training set for re-training. Tracking errors using this new model are lower and images which this model performs poorly on can be found and the whole process is repeated. The error histogram after different numbers of training rounds is shown in Fig. 2. We found that re-training twice significantly reduced tracking error while not significantly increasing the dimensionality of the model. The final model is built from 71 training images, resulting in an AAM controlled by 17 parameters, which together with their first time derivatives are used in the CAT model (Fig. 3).

When animating a face it is useful to be able to control certain actions such as eye blinks and head rotation. This is difficult with a standard AAM since the modes in a standard AAM have no physical meaning. We therefore train an AAM in which one mode corresponds to blinking and two modes to head rotation. We find the shape components that model head pose by recording a training sequence of head rotation with a fixed neutral expression. The pose components are removed in each training shape to obtain pose normalised training shapes, which model only facial deformation, see [48]. Analogously a mode for eye blinking is found by using sample frame from the same blink event. A further extension is training a model in which the upper and lower regions of the face are controlled independently. This builds a model in exactly the same way as the previous section except that modes only deform specific areas of the model. In [48] it is shown that these extensions improve the synthesis quality as measured in terms of maximum *L*_2_ tracking errors, as well as in user preference (Table 1).

### 3.4 Synthesis interface

**Fig. 4:**
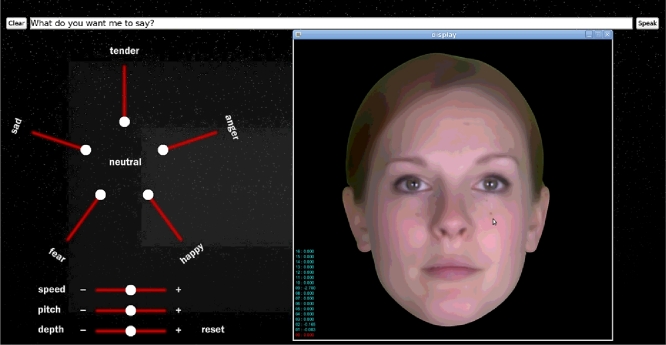
Screenshot of interface for synthesising with XpressiveTalk. The interface allows for inputting text and setting the values of the expression parameters which are used to create the animation of the talking avatar.

Fig. 4 shows the XpressiveTalk synthesis interface that was used to create samples for the current study. The user types in the text in the text box, and the desired emotion can be specified by adjusting the position of the sliders. Upon clicking “Speak”, the synthesis engine is run and a synthesised video file is produced and played back. When the sliders are all in the inner-most position (0%), the system assumes a zero-weight for all non-neutral emotions, and neutral speech/video is produced. Pure emotions can be synthesised with various degrees by moving the slider for one emotion to a non-zero position. A combination of emotions is also possible, by setting the sliders for multiple emotions to non-zero positions.

## 4 Method

### 4.1 Participants

The ASC group comprised 40 adolescents and adults (23 female, 17 male) aged 19–63 years, recruited from the Cambridge Autism Research Database (CARD) website [50]. All participants with ASC who register to take part in online research through this website have been formally diagnosed by a clinician according to DSM-IV criteria [51]. In addition, all participants completed the Autism Spectrum Quotient (AQ) [52] to indicate the number of autistic traits of participants in the ASC compared to the typical control group. The control group comprised 39 adolescents and adults (32 female, 7 male) aged 16–63 years, recruited from a separate research website for the general population without ASC diagnosis [53]. Groups were matched on age, but not gender (*X*^2^(1) = 5.6, *p* = 0.02), however, there was no significant effect of gender on task performance in the control group.

### 4.2 Materials

The real face condition consisted of 20 videos of a female actress speaking four neutral sentences, (a) ‘the actual number is somewhat lower’; (b) ‘the beach is dry and shallow at low tide’; (c) ‘the fan whirled its round blades softly’; and (d) ‘we don’t have any choice’), each in five different emotional tones; happy, sad, angry, afraid and neutral. The XpressiveTalk condition consisted of 20 videos synthesised using the interface described in Section 3.4, in the same four neutral sentences, each synthesised in the same five emotional tones, each with the weight for the respective emotion set to 100% and other emotions set to 0%, in the face and voice domains. These basic emotions were chosen to be included from the interface, excluding tender, as these had been utilised in previous research studies (e.g. [20], [21]), and could be of particular benefit to adults with ASC who have difficulties recognising negative basic expressions such as fear and sadness.

### 4.3 Procedure

Participants were invited to complete an emotion recognition study through a secure website, and provided their consent to take part electronically. They then completed a brief registration process (age, gender, ASC diagnosis and subtype, any family members with ASC diagnosis, any other diagnoses), and completed the AQ. They were then shown videos of emotion expressions performed by the original actress (real face condition), and synthesised emotion expressions through XpressiveTalk. Each emotion was expressed in four neutral sentences for both the real and synthesised faces, to control the context of the sentence between conditions. In total there were 100 synthesised videos and 100 real-face videos, presented in a random order.

After seeing each video, participants were asked to; (a) choose which emotion they thought it was from five options (happy, sad, angry, afraid and neutral); (b) rate their preference (‘How much did you like this face?’); and (c) rate how realistic they thought it was (‘How real did you think the face was?’). Participants had two weeks to complete the task.

## 5 Results

### 5.1 Analysis approach

A General Linear Model approach is used in the analysis of behavioural results from the user study. Analysis of Variance (ANOVA) are used to explore differences in the percentage correct emotion inferences, preference and realism ratings, for each emotion (happy, sad, angry, afraid, neutral), in each group (ASC and typical control), and condition (real face and XpressiveTalk). Significant interactions between variables, suggesting that the pattern of results is different between variables (e.g. emotion recognition accuracy may improve for certain emotions between conditions), are explored further using simple main effects analysis. Significant main effects for variables involving more than one level (e.g. in the case of five emotion types), are explored further using Bonferroni corrected t-tests, with *p* values corrected for the increase in chance of finding a significant effect when undertaking multiple comparisons (see [54], [55]).

### 5.2 Emotion recognition

Tables 2 and 3 show the confusion matrices for participants’ emotion inferences in the typical control and ASC groups in each condition respectively. Both groups appear to provide more correct than incorrect emotion inferences for both the real and XpressiveTalk conditions. However, those with ASC appear to be less accurate overall than typical controls. Participants in both groups also appear to be less accurate when inferring happy and angry from XpressiveTalk compared to the real face.

A three way mixed ANOVA compared group (ASC, typical control), condition (real, XpressiveTalk), and percentage of correct emotion responses (happy, sad, angry, afraid, neutral). Participants with ASC (mean = 70%) were significantly less accurate than typical controls (mean = 83.4%) (*F*(1,77) = 21.7, *p* < 0.001). There was a significant main effect of emotion (*F*(4,308) = 11.25, *p* < 0.001). Bonferroni corrected t-tests showed that participants were significantly more accurate when inferring neutral than happy, angry, fear and sad (all *p* < 0.001). There was a significant interaction between condition and emotion (*F*(4,308) = 33.5, *p* < 0.001), suggesting that the pattern of correct emotion inferences was significantly different in each condition. Simple main effect analyses showed that participants were significantly less accurate at inferring angry (*F*(1,77) = 89.2, *p* < 0.001) and happy (*F*(1,77) = 52.3, *p* < 0.001), and significantly more accurate at inferring sad (*F*(1,77) = 14.8, *p* < 0.001) from XpressiveTalk compared to the real face. There were no significant differences in accuracy for recognition of fear or neutral emotions from XpressiveTalk compared to the real face (Table 4).

### 5.3 Preference ratings

Table 5 shows the preference ratings for each emotion in each group and condition. The ASC group gives lower preference ratings than the typical control group overall. In both groups, negative emotions (sad, angry, afraid) are rated as less preferred than happy. Synthesised sad and neutral emotions appear to be rated as more preferred than real faces, whereas happy, angry and afraid emotions are rated as less preferred in the XpressiveTalk than the real face condition. A three way mixed ANOVA compared group (ASC, typical control), condition (real, XpressiveTalk), and mean preference ratings for each emotion (happy, sad, angry, afraid, neutral). Typical controls (44.2) showed a significantly higher preference for faces than individuals with ASC (34) (*F*(1,77) = 5.6, p = 0.02). There was a significant main effect of emotion (*F*(4,308) = 24.9, *p* < 0.001). Bonferroni correct t-tests showed that neutral and happy faces had significantly higher preference ratings than sad, angry and afraid (all *p* < 0.05). There was a significant interaction between condition and emotion (*F*(4,308) = 43.5, *p* < 0.001). Simple main effect analyses showed that both group’s preference ratings were significantly lower for happy (*F*(1,77) = 54, *p* < 0.001), angry (*F*(1,77) = 13.8, *p* < 0.001) and fear (*F*(177) = 33.8, *p* < 0.001) for XpressiveTalk compared to the real face. Yet, for the emotions sad (*F*(1,77) = 60.5, *p* < 0.001) and neutral (*F*(1,77) = 36.1, *p* < 0.001) the preference rates were significantly higher in the XpressiveTalk face, compared to the real face.

### 5.4 Realism ratings

Table 6 shows the realism ratings for each emotion in each group and condition. The ASC group appears to give lower realism ratings than the typical control group overall. A three way mixed ANOVA compared group (ASC, typical control), condition (real, XpressiveTalk), and mean realism ratings for each emotion (happy, sad, angry, afraid, neutral). There was a significant main effect of emotion (*F*(4,308) = 6.4, *p* < 0.001). Bonferroni corrected t-test showed that fear was rated as significantly less real than sad, and angry and neutral (all *p* < 0.01). There was a significant interaction between condition and emotion (*F*(4,308) = 95.2, *p* < 0.001). Simple main effect analyses showed that the synthesised happy (*F*(1,77) = 97.3, *p* < 0.001), angry (*F*(1,77) = 85.8, *p* < 0.001) and afraid (*F*(1,77) = 6.4, p = 0.014) emotions were rated as significantly less realistic compared to the real faces. Synthesised sad (*F*(1,77) = 67.2, *p* < 0.001) and neutral (*F*(1,77) = 169.1, *p* < 0.001) emotions were rated as significantly more realistic than the real faces.

## 6 Discussion

In this study we present a method for generating a near-videorealistic avatar, which can convert input text into expressive speech and face, and discussed its potential as an assistive technology to improve emotion processing skills and social attention in adults with ASC. Our results show that neutral and sad expressions synthesised through XpressiveTalk were convincing; both adults with and without ASC showed significantly increased accuracy from XpressiveTalk (compared to the footage of the real face), and rated these expressions as significantly preferred and more realistic. There was no significant difference in recognition accuracy of fear between XpressiveTalk and the real face. However, participants were significantly less accurate when inferring synthesised happy and angry expressions through XpressiveTalk compared to the real face, and rated these expressions as significantly less preferred and realistic. Thus, the synthesised happy and angry faces through XpressiveTalk appeared to be less expressive, and more difficult to infer emotions from than those portrayed by the original actress. This is also reflected by the fact that synthesised happy and angry expressions tended to be confused more with neutral faces for XpressiveTalk than the real face.

Our results also show emotion recognition difficulties in adults with ASC for the real face, and XpressiveTalk, reflecting results of previous studies, where more realistic emotions, involving a moving talking face, tend to show emotion recognition difficulties in adults with ASC [12], [13], [14]. This result shows the benefit of utilising these kinds of more naturalistic, dynamic stimuli, which more closely match the emotion expressions encountered in everyday life. Additionally, the fact that the synthesised emotions presented at high (100%) intensity through XpressiveTalk were sensitive enough to detect emotion recognition difficulties in high functioning adults with ASC, means that this interface is potentially useful as an intervention tool, where there is room for performance to improve through use of the interface.

Both groups of participants still performed well above chance level for recognition of emotions from XpressiveTalk and the original actress, even in the case of synthesised happy and angry faces. Adults with ASC also showed significantly reduced preference for faces (regardless of stimulus type), compared to typical controls overall, consistent with previous studies showing avoidance of people and faces in ASC [38]. These results are consistent with previous research showing reduced preference, engagement and ability to process emotions in ASC (e.g. [14], [38], [41], [42], [43]).

However, adults with ASC were able to engage with the interface, and showed a similar pattern of preference and judgment of realism to typical controls. Participants with ASC who took part in the study also commented that the use of an avatar, as opposed to a real person, created a sense of anonymity and distance, which made it easier to look into the face and in particular the eyes of the face. This reflects the results of previous studies which have shown that interactive technology has the potential to provide a safe and predicable learning opportunity for adults with ASC, which does not have the same anxiety provoking nature as social situations in the real world [3], [4]. Hence, XpressiveTalk could provide an opportunity for adults with ASC to access and engage with the social world, through non aversive means. We aim to explore in future whether repeated exposure and experimentation with XpressiveTalk in adults with ASC, improves their ability to attend to and recognize emotions from XpressiveTalk, the original actress, and others’ emotion expressions.

In order to maximize the chances of an intervention to be useful to adults with ASC, the expressiveness of synthesised faces needs to have a similar, if not higher level of signal clarity than real faces. Adults with ASC have particular difficulty interpreting emotions of low signal clarity (e.g. [16]). A particular strength of XpressiveTalk is that the signal clarity of the emotion expressions can be systematically manipulated (mixing emotions of differing levels of intensity) by the participant throughout the intervention. This provides the participant engaging with the interface to experiment with a large emotion space and full spectrum of signal clarity. The participant could therefore gradually increase the difficulty level of the emotions by reducing the signal clarity of these as they improve. In the current study, we employed simple emotions at 100% intensity to compare with the original actress, in order to ascertain how the level of signal clarity for synthesised faces compared to the real actress. At this high intensity, synthesised neutral and sad expressions appear to have significantly higher signal clarity that the original actress, whereas happy and angry faces appeared to have significantly lower signal clarity than the original actress.

## 7 Conclusion

In conclusion, new interactive technologies are a promising intervention tool to improve emotion processing and attention skills in adults with ASC. This study presents a method for generating a video of expressive speech, which can be manipulated by the user, to generate a wide array of emotions differing in their level of intensity and complexity. We demonstrate that adults with ASC show evidence of greater engagement with the synthesised compared to the real faces of the original actress. Both adults with and without ASC also show a similar pattern of recognition and realism ratings for synthesised as compared to real faces. In particular, synthesised neutral and sad faces are recognised more accurately than the real face, suggesting these synthesised expressions have significantly higher signal clarity than the original actress. Synthesised happy and angry faces require improvement in their signal clarity, in order to ensure that adults with ASC can begin the intervention at a high level of signal clarity, and gradually lower this and thus gradually increase the complexity of the emotions at their own pace.

## Figures and Tables

**Table 1:** Participant characteristics. Autism Quotient (AQ) scores are missing for three participants in the typical control group.

	ASC group	Control group	
	(*N* = 40)	(*N* = 39)	
	Mean ± S.D.	Mean ± S.D.	
	(Range)	(Range)	*t*-test result
Age (years)	40.9 ± 13.2	43.7 ± 14.8	*t*(77) = .9, *p* = .37
	(19–63)	(16–63)	
AQ	40.4 ± 6.2	17.8 ± 10.4	*t*(74) = .11.4, *p* < .001
	(19–49)	(3–42)	

**Table 2:** Confusion matrices showing the percentage of emotion inferences for real faces and XpressiveTalk in the typical group.

		Real face					XpressiveTalk				
		Correct emotion					Correct emotion				
		Happy	Sad	Angry	Afraid	Neutral	Happy	Sad	Angry	Afraid	Neutral
Emotion response	Happy	87.2	0.0	0.0	0.0	1.9	66.0	0.0	1.3	0.0	1.9
	Sad	0.0	74.4	0.0	5.8	3.2	0.0	85.9	0.6	10.9	0.0
	Angry	1.3	0.0	94.9	2.6	1.9	1.9	0.0	64.7	1.9	3.2
	Afraid	0.6	22.4	1.9	89.1	1.9	15.4	12.2	15.4	85.9	0.0
	Neutral	10.9	3.2	3.2	2.6	91.0	16.7	1.9	17.9	1.3	94.9

**Table 3:** Confusion matrices showing the percentage of emotion inferences for real faces and XpressiveTalk in the ASC group.

		Real face					XpressiveTalk				
		Correct emotion					Correct emotion				
		Happy	Sad	Angry	Afraid	Neutral	Happy	Sad	Angry	Afraid	Neutral
Emotion response	Happy	77.5	0.0	1.9	0.0	2.5	43.8	0.0	2.5	0.0	6.9
	Sad	0.0	60.0	0.0	13.8	4.4	5.0	79.4	2.5	11.3	3.8
	Angry	4.4	1.3	86.3	5.6	2.5	1.3	0.0	53.1	6.3	5.0
	Afraid	2.5	20.6	2.5	68.8	3.1	14.4	13.8	19.4	60.0	0.6
	Neutral	15.6	18.1	9.4	11.9	87.5	35.6	6.9	22.5	22.5	83.8

**Table 4:** Confusion matrices showing the percentage of emotion inferences for real faces and XpressiveTalk (typical and ASC groups combined).

		Real face					XpressiveTalk				
		Correct emotion					Correct emotion				
		Happy	Sad	Angry	Afraid	Neutral	Happy	Sad	Angry	Afraid	Neutral
Emotion response	Happy	82.3	0.0	0.9	0.0	2.2	54.7	0.0	1.9	0.0	4.4
	Sad	0.0	67.1	0.0	9.8	3.8	2.5	82.6	1.6	11.1	1.9
	Angry	2.8	0.6	90.5	4.1	2.2	1.6	0.0	58.9	4.1	4.1
	Afraid	1.6	21.5	2.2	78.8	2.5	14.9	13.0	17.4	72.8	0.3
	Neutral	13.3	10.8	6.3	7.3	89.2	26.3	4.4	20.3	12.0	89.2

**Table 5:** Preference rating for each emotion in the ASC and typical control group, in the real face and XpressiveTalk conditions.

	Real face					XpressiveTalk				
	Happy	Sad	Angry	Afraid	Neutral	Happy	Sad	Angry	Afraid	Neutral
ASC	44.6	22.8	33.3	39.2	34.9	28.8	39.3	24.7	28.7	43.9
Typical control	58.1	34.0	41.4	46.1	44.8	40.1	49.2	32.3	38.4	57.1
Total	51.3	28.4	37.3	42.6	39.8	34.4	44.2	28.5	33.5	50.4

**Table 6:** Realism rating for each emotion in the ASC and typical control group, in the real face and XpressiveTalk conditions.

	Real face					XpressiveTalk				
	Happy	Sad	Angry	Afraid	Neutral	Happy	Sad	Angry	Afraid	Neutral
ASC	61.6	36.3	64.0	47.5	32.2	32.4	63.9	36.8	40.3	62.6
Typical control	69.4	44.0	70.0	53.4	37.6	38.7	70.7	40.2	50.3	73.3
Total	65.5	40.1	66.9	50.4	34.9	35.5	67.2	38.5	45.2	67.9
